# Editorial: Comparative biology of red blood cells

**DOI:** 10.3389/fphys.2022.1103647

**Published:** 2022-12-14

**Authors:** Anna Bogdanova, Lars Kaestner

**Affiliations:** ^1^ Red Blood Cell Research Group, Institute of Veterinary Physiology, Vetsuisse Faculty, University of Zurich, Zurich, Switzerland; ^2^ Theoretical Medicine and Biosciences, Campus University Hospital, Saarland University, Homburg, Germany; ^3^ Dynamics of Fluids, Experimental Physics, Saarland University, Saarbruecken, Germany

**Keywords:** red blood cells, animal models, adaptation, deformability, sickle cell disease, metabolism, K-Cl contransporter, hibernation

Our interest in the broad diversity of red blood cell (RBC) sizes, composition, gas transport strategies, rheological properties, and metabolism across the animal kingdom is fueled by several reasons. Biologists follow the developmental and evolutionary aspects of this RBC diversity that enables the hosts to survive and populate habitats that vary in O_2_ concentration and nutritional abundance matching energy supply and demand (Nikinmaa, 1990). Translational researchers view animals as models to study human diseases, although numerous studies urge for care in translation of the findings obtained using rodents or other mammals as well as insects, nematodes and fishes to the human patients (Smith, 1981; van der Worp et al., 2010; Robinson et al., 2019; Doncheva et al., 2021; Mukherjee et al., 2022). Finally, veterinarians are focused on treatment of diseases of their animal patients associated with RBC abnormalities (Brooks et al., 2022). These three groups of scientists do not speak “the same language” and the journals publishing the findings in clinical hematology do recognize the publications dealing with comparative aspects of RBC physiology. This topic was a challenging attempt to fuse the interests of both sides, applying the methodological approaches developed for human RBC to the animal cells. The four papers forming this volume meet a common ground touching upon various parameters in control of RBC rheology and life-span. These parameters include the regulation of cell hydration and volume (Lu et al.), oxygenation of hemoglobin (Bertolone et al.; Lu et al.; Man et al.), metabolism and antioxidative defense systems (Klichkhanov et al.; Bertolone et al.). In their study Kichkhabnov et al. monitor the changes in production of NO and in the markers of oxidative stress and antioxidant enzymes in RBCs of the ground squirrels while active, during hibernation, and during the arousal phase when their body temperature and metabolic activity undergo massive and rapid transition. Robust antioxidative defense mechanisms in RBCs of these animals are described allowing to avoid hemolytic crisis despite the increase in oxidative load during arousal. The inability of guinea pigs to produce their own ascorbate makes them dependent on its dietary intake and prone to scurvy development. Another dark side of this metabolic feature, as described by Bertolone et al., is higher susceptibility of RBCs of guinea pigs to blood storage lesions.

Sickling of RBC is a phenomenon that most of us associate with human sickle cell disease. However, this mechanism has been evolved much earlier and is used to combat blood infection by fishes, reptiles, and other mammals (Steinberg, 2019). In humans, as well as in other species, hemoglobin aggregation is not occurring in all cells, but in a fraction of them. In their work Man et al. introduce an OcclusionChip that may detect the abundance of cells in the fraction that loses deformability and may initiate thrombosis. They evaluate the sensitivity of this approach using blood of healthy people and sickle cell disease patients comparing this with the outcome of oxygen gradient ektacytometry. The use of the OcclusionChip for animal blood samples with “sickling red blood cells” should provide important information on the impact of sickling of RBCs of different species on their rheological properties. Regulation of the K-Cl cotransporter (KCC) contributes to the maintenance of RBC volume, and its hyperactivation in cells of patients may induce aggregation of sickle hemoglobin (HbS) and promote the loss of deformability due to the irreversible sickling. The study of Lu et al. explores the role of bicarbonate, pH, and urea levels in plasma, as well as hemoglobin oxygenation, in regulation of the KCC function in low K^+^ sheep RBCs. The obtained data shed light on some of the underexplored aspects of dehydration mechanisms in human sickle cell disease patients.

In line with the publications of this Research Topic we hope to further stimulate translational RBC research. Neither is it explicidly limited to the utilization of transgenic (mouse) models to mimic human diseases with all the well-known associated challenges nor to use animals as disease models for practical reasons, e.g. (Thomas et al., 2001), but the use of natural genetic and functional heterogeneity of RBCs from various species such as the elliptic camel RBCs as models for elliptocytosis (Smith et al., 1979; Amin and Sirs, 1985; Windberger et al., 2018; Baier et al., 2021), naturally sickling RBCs of deer, sheep, goat, genets and mongoose, Gulf toadfish, iguana, snakes, or fishes for sickle cell disease (Steinberg, 2019), cow and sheep RBC as models for lipid alterations (Engen and Clark, 1990; Nouri-Sorkhabi et al., 1996; Ivanov, 2007), and cattle (Inaba et al., 1996; Jay, 1996) or even lamprey (Cameron et al., 2000) RBCs as a model of Band 3 protein deficiency. We are looking forward to it.
